# Metabolic syndrome cardiovascular risk prevention by omega-3 polyunsaturated fatty acids

**DOI:** 10.1093/ehjopen/oead115

**Published:** 2023-11-12

**Authors:** Magnus Bäck, Roberto Latini

**Affiliations:** CArdiovascularREsolution of INflammation to promote HEALTH (CARE-IN-HEALTH) Consortium; Department of Cardiology, Heart and Vascular Center, Karolinska University Hospital, Stockholm, Sweden; Department of Medicine Solna, Karolinska Institutet, Stockholm, Sweden; Nancy University Hospital, University of Lorraine and INSERM U1116, 9 avenue de la Foret de Haye, BP 20199, 54505 Vandoeuvre les Nancy Cedex, France; CArdiovascularREsolution of INflammation to promote HEALTH (CARE-IN-HEALTH) Consortium; Department of Acute Brain and Cardiovascular Injury, Istituto di Ricerche Farmacologiche Mario Negri—IRCCS, Via Mario Negri, 2, 20156 Milano, Italy

**This editorial refers to ‘Effectiveness of icosapent ethyl on first and total cardiovascular events in patients with metabolic syndrome, but without diabetes: REDUCE-IT MetSyn’, by M. Miller *et al*. https://doi.org/10.1093/ehjopen/oead114.**

Studies of cardiovascular (CV) preventive strategies with high-dose omega-3 polyunsaturated fatty acids (n-3 PUFA) in subjects with hypertriglyceridaemia have generated differential results, which may relate to PUFA formulations, dosing and relative content of the two n-3 PUFAs docosahexaenoic acid (DHA) and eicosapentaenoic acid (EPA).^1^ The n-3 PUFA-induced triglyceride lowering has also been suggested to be independent of the either beneficial or neutral cardiovascular outcomes in hypertriglyceridaemic subjects.^1,2^ Importantly, hypertriglyceridaemia is one of the criteria for metabolic syndrome (MetSyn) combined with increased waist circumference and blood pressure as well as high glucose and low HDL levels.

In this issue of *European Heart Journal Open*, a pre-specified MetSyn subgroup analysis, including 35% of the 8179 patients enrolled in REDUCE-IT,^3^ showed an applicability of the beneficial effects of the EPA formulation icosapent etyl (IPE) on the primary composite endpoint in subjects with MetSyn without diabetes.^3^ Among the endpoint components, myocardial infarction, coronary revascularization, and unstable angina requiring hospitalization were significantly lower in IPE vs. placebo in line with results obtained in the total study population.^2^ In contrast, the reduced risk for cardiovascular death and stroke in REDUCE-IT^2^ were not significant in the REDUCE-IT MetSyn subgroup,^3^ which may illustrate the increased MetSyn risk profile. Finally, IPE increased hospitalization for either atrial fibrillation or flutter events in the MetSyn subgroup and did not alter risk of new onset diabetes.^3^

The authors conclude that these results support that IPE reduces the high CV risk MetSyn phenotype despite lacking robust effects on MetSyn components apart from triglyceride lowering.^3^ Interestingly, an analysis of the individual MetSyn components (except triglycerides) revealed that IPE significantly reduced primary endpoint risk independently of the fulfilment of the waist circumference and elevated fasting glucose MetSyn criteria at baseline.^3^ In contrast, the IPE-induced effects were less pronounced and non-significant in the absence of blood pressure and HDL criteria.^3^

One of the challenges in the interpretation of variable results of cardiovascular outcomes in clinical trials of omega-3 PUFA in addition to differences in doses, formulations and the EPA/DHA content, is identifying factors distinguishing responders from non-responders to omega-3 PUFA treatment.^1^ Differential effects of IPE depending on the MetSyn criteria^3^ opens up for future studies of blood pressure and HDL as possible factors to take into consideration in a personalized dimension for n-3 PUFA indications in cardiovascular prevention. Other possible underlying factors to a personalized response to n-3 PUFA intake and supplementation include genetic variations in fatty acid metabolism. For example, polymorphisms within the fatty acid desaturases (FADS) 1 and 2 FADS loci alter desaturase activity, which influence PUFA levels following n-3 PUFA supplementation or dietary intake and associate with measures of cardiovascular risk.^4^

Omega-3 PUFAs also serve as the substrate for bioactive lipid mediators. The release of EPA from IPE provides the precursors for biosynthesis of resolvin E1, which mediates an active resolution of inflammation.^1^ The contribution of low-grade non-resolving chronic inflammation in atherosclerotic cardiovascular disease has been well established. MetSyn represents an increased inflammatory state and median plasma concentration of hsCRP were slightly elevated (1.9 mg/L) in the REDUCE-IT MetSyn population.^3^ Previous reports on biomarkers in REDUCE-IT indicated minor changes in hsCRP levels by IPE treatment.^5^ No prospective data were provided for the effects of treatment on hsCRP in subjects with MetSyn, and potential changes in the MetSyn components measures were also not reported in the REDUCE-IT MetSyn subgroup analysis.^3^

The results presented in this issue of *European Heart Journal Open* that IPE reduced cardiovascular events in subjects with MetSyn^3^ extends the knowledge on omega-3 PUFA in cardiovascular prevention. The observations on IPE cardiovascular risk prevention according to MetSyn criteria add new clues to the genetic and inflammation paths that are currently followed in the quest for identifying responders and non-responders to omega-3 PUFA cardiovascular prevention.

## Data Availability

No new data were generated or analysed in the preparation of this editorial.
